# The service control tower: dilemmas, decisions and a reference architecture for maintenance of (high-value) assets

**DOI:** 10.1007/s10257-025-00705-6

**Published:** 2025-05-24

**Authors:** Rogier Harmelink, Engin Topan, Jos van Hillegersberg

**Affiliations:** 1 Industrial Engineering and Business Information Systems, School of Behavioural and Management Sciences, University of Twente, Drienerlolaan 5, 7500 AE Enschede, Netherlands; 2 Jheronimus Academy of Data Science, Sint Janssingel 92, 5211 DA ’s-Hertogenbosch, Netherlands

**Keywords:** Service control tower(s), Dilemmas, Inter-organizational system, Maintenance-oriented architecture, Reference architecture

## Abstract

Complex supply chains require coordination efforts from supply chain partners. The concept of the service control tower plays an essential role in the transition towards Logistics 4.0 and coordination in supply chains. This paper analyses the concept of the (service) control tower. We find different (service) control tower applications in the literature. Some are focused on a specific domain (e.g., transportation and pharmaceutical logistics), while others have a more generic approach. We review available architectures and determine strengths and gaps. Additionally, we find that the construction of inter-organizational systems is complex. We explore the process of collaboration in four phases. Next, we define the service control tower and recognize four levels that organizations construct to create the system. However, in an inter-organizational context, strategic interests cause conflict and result in different technical and business dilemmas. We identify several dilemmas based on experts’ inputs during workshops with companies in constructing a service control tower. Most dilemmas occur in an early stage of development and are often related to the system’s governance, data sharing and IT integration. Finally, we provide potential users, developers and researchers of (service) control towers with a maintenance-oriented architecture for service control towers.

## Introduction

The term control tower refers to an artefact that controls air traffic in and surrounding airports. In the past couple of years, the term has become more famous for integrating IT systems and optimization in a supply-chain context. The control tower analogy has now found its way into logistics and supply chains for process visibility and governance of inter-organizational relations. From a historical perspective, the concept of the control tower as an information system links back to the origin of the inter-organizational system, as first described by Kaufman (1966). Later, Barrett and Konsynski (1982) defined an inter-organizational system as ’a general term referring to systems that involve resources shared between two or more organizations’. A key component in an inter-organizational system is data sharing based upon computer technology (Suomi 1992). An inter-organizational system can reduce costs, increase productivity, and the product market strategy (Barrett and Konsynski 1982). There are some well-known implemented inter-organizational systems. Examples are AIS for vessel identification, SWIFT for financial transactions and eIDAS for electronic identification of SMEs in Europe. However, inter-organizational systems are also beneficial for supply chain management (Humphreys et al. 2001). At the same time, the examples of inter-organizational systems show similarity to a control tower in a broader context. This paper focuses explicitly on control tower systems that enable visibility and control in supply chains to reach companies’ (individual) business goals.

Organizations use control tower applications to optimize their transportation and logistics processes. To put the control tower in a historical perspective, the use of IT in an intra-organizational (i.e., within an organization) has a rich history. A big turnaround in the use of IT in organizations was the introduction of Enterprise Resource Planning (ERP) in the 1990s. ERP is an umbrella term for software applications that integrate business management processes due to a lack of interfacing to older business software (Jacobs and Weston Jr. 2007). To remain competitive, companies use ERP as an effective and necessary tool for managing processes (Spathis and Constantinides 2003). In recent decades, ERP has increasingly integrated with Supply Chain Management Software (SCM) (Tarn et al. 2002). Organizations integrating ERP with SCM systems have a significantly higher business performance (Wieder et al. 2006). Therefore, the focus within organizations is also shifting towards a broader supply chain driven context. The control tower concept for use in a supply chain context aims to bridge that gap.

In this paper, we explore the concept of the Service Control Tower (from here on SCT) in SCM from an intra- and an inter-organizational perspective. We build upon the work done by Topan et al. (2020), define the SCT explicitly and expand the focus on that in three dimensions (i.e., defining the system, introducing its dilemmas and constructing a reference architecture). Organizations have increased interest in these specific control towers as they could deliver optimal service concerning certain activities. The case of interest discussed in this paper concerns the maintenance of maritime assets. The SCT could help organizations reduce maintenance costs, increase resilience in the supply chain, and increase the availability of their assets. The SCT could play a vital role in solving the current issues faced in the supply chain. However, setting up such a SCT environment is not trivial. In this paper, we will discuss the most critical elements regarding construction.

The paper’s outline is as follows. In Section 2, we review the state-of-the-art literature on CTs. Section 3 reflects upon the concept of the (S)CT and explains how to approach the generalization of the concept, including a methodological approach. Section 4 defines the SCT and describes the different steps (i.e., levels) organizations must follow to construct such a system. We describe different dilemmas organizations face in setting up such a system in Section 5. Additionally, we introduce the collaboration tool by Dinalog (Dutch Institute for Advanced Logistics^1^), a tool organizations could use to collaborate in a supply chain. Next, Section 6 discusses a reference architecture for an SCT, i.e., a blueprint for organizations containing the components and an architecture for data and performance measurements. Finally, we conclude this paper in Section 7 with the main conclusions and a discussion of the implications.

## Literature on (service) control towers

Control towers in a supply-chain context serve various goals. Additionally, a network of CTs is named differently as a cross chain control center (4C), which is used and referred to by multiple papers (e.g., Trzuskawska-Grzesińska 2017, te Lindert 2013, Dalmolen et al. 2015). Examples of CTs are integrated logistics CT (e.g., in the off-shore Mohammad and Mohd Azani 2018), transportation CT (e.g., Baumgrass et al. 2014) and a CT for rail freight services (e.g., Milenković et al. 2019). However, a new type of CT covers service logistics, namely the SCT. The SCT has a specific focus in the service logistics domain, defined by Davis and Manrodt (1991) as ”the management of activities which respond to customers on an individual basis". The critical difference with regular logistics is that the organization that offers a product does not only look at material flows but incorporates the coordination and interaction with the customer to respond to individual needs. So, the focus shifts from selling a product to integrating this with maintenance, training, repairs, and additional services.

A range of definitions also covers the multiple applications of (S)CTs (Trzuskawska-Grzesińska 2017) originating from white papers covering aspects of the CT and define the CT as applications for enabling supply chain visibility, achieving strategic objectives and improving decision making. Verma et al. (2020) define the CT as ’a concept which comprises integrated supply chain and decision-making related to its needs to be fulfilled by the system capable of real-time tracking, alerts, visualization, and giving quick solutions is achievable.’ Milenković et al. (2019) define the CT for rail freight services as ’an information-sharing platform that will support planners in supply chain optimization and fulfill the shipper’s requirements for real-time visibility in whole transport chain’. Alias et al. (2014b) use the definition that: ’control towers are decision-support systems merging different data streams from various subordinate levels and displaying the consolidated information at a higher level for monitoring and control of processes while pursuing the goal of optimal process operation.’.

The three definitions given above have some overlap but also some distinct features. All three relate to process optimization or controlling an (integrated) supply chain. Both Verma et al. (2020) and Milenković et al. (2019) discuss real-time visibility or tracking as a key to this. However, on the contrary, Alias et al. (2014b) does not discuss real-time as an explicit requirement but states that data comes from different levels and merges at a higher level. Therefore, one could doubt the need for real-time data or information. In the case of vessels, data is often unavailable in real-time but travels via AIS (analogue signals), 5G, or satellite connections with delay. As a result, one might ask if such a requirement is realistic and necessary for a CT system to function correctly.

Another striking aspect is that Milenković et al. (2019) calls the CT ’an information sharing platform’, while Alias et al. (2014b) talks about ’a decision-support system’. These have two completely different working-outs, potentially complicating a CT solution’s generalizability. ’Decision-support’ suggests that the system cannot fully operate independently, while in more advanced CT cases, this might be possible and beneficial. An ’information-sharing platform’ might imply that any platform that shares information fits the perspective of a CT, but this is not often the case. We, therefore, limit ourselves in this paper to the three main points discussed by Trzuskawska-Grzesińska (2017); a CT should enable supply chain visibility, achieve strategic objectives (i.e., with servitization, in our case) and improve decision-making.

One of the other main questions is the business rationale behind developing a(n) (S)CT solution. Why are organizations interested in these IT artefacts, and how do they expect to increase their operational efficacy by applying such a system? Alias et al. (2015) construct a Business Model Canvas exploring the reasons for developing and adopting a CT solution. The principal value propositions are better decision-making, visibility in the supply chain, gains in efficiency and cost reduction (Alias et al. 2015). However, these propositions are generic for organizations that act in a business environment. The vision surrounding the (S)CT artefact is that it should be easier to connect to this artefact as ’one integrates all’ solutions for specific supply chain processes. An additional value proposition is that organizations must invest in the software and hardware supporting the CT. These essential cost aspects could be turned into a revenue model by offering the (S)CT solution as a service (Alias et al. 2015).

(S)CTs are constructed more often in an intra-organizational context. In the inter-organizational context, multiple issues hinder the implementation of such systems. For example, te Lindert (2013) mentions three problems: The lack of a fair sharing mechanism, trust and intelligent IT tools. Regarding the aspect of intelligent IT tools, Dalmolen et al. (2015) mention essential requirements for CT solutions: modularization of services, products and processes, and capabilities for coordination, collaboration, quick connect, relationship management and risk management. On the contrary, fair sharing mechanisms and the lack of trust lack coverage.

In the literature, we find multiple papers describing a control tower architecture. For example, Shou-Wen et al. (2013) visualize the concept of a ’Supply Chain Information Control Tower’. Their five-layered architecture described the interactions between layers and included technologies used within them. On the other hand, Alias et al. (2014a) describes a general CT architecture based on the main functions: monitoring and control. They base them on a so-called MAPE-K (Monitor, Analyze, Plan, Execute and Know) loop, which is generically applicable in any CT environment. On the contrary, their architecture is not an IT architecture.

Baumgrass et al. (2014) portrays an example of an architecture of a CT focused on transportation. They consider different software components. However, how they fit a specific business requirement must also be clarified. Hofman (2014) describes a logistics CT’s components and shows how they interfere without providing the details on the interfaces and the software needs. Finally, Rustenburg (2016) takes a CT approach to spare parts planning. The architecture is maintenance-oriented because of the focus on spare parts. However, the IT architecture is based on Microsoft software, making it vulnerable to vendor lock-in.

Liotine (2019) focuses on a functional architecture for pharmaceutical logistics but needs more insights into the exact IT components. Last, we discuss an architecture for processes in the SCT by Topan et al. (2020). They discuss an empirically founded process architecture that still needs supporting IT architecture. Topan et al. (2020) is, to our knowledge, the only paper that reflects upon (partial) implementations of SCTs. Although that Topan et al. (2020) focuses on a centralized SCT, they recognize that the supply chain network served is highly decentralized.

In conclusion, we see in the literature multiple architectures, summarized in Table 1. Some discuss a specific focus of CT (e.g., Liotine 2019, Hofman 2014,Baumgrass et al. 2014, Rustenburg 2016). Others are more generic (e.g., Alias et al. 2014b, Shou-Wen et al. 2013, Topan et al. 2020). Next, we see that some focus on IT but lack the IT architecture. In this paper, we complement the work of Topan et al. (2020) by creating a reference architecture and explaining potential dilemmas and design decisions in developing an SCT. Topan et al. (2020) reviews multiple applications for SCTs in an after-sales support context. This paper focuses mostly on the purpose and architecture of an SCT.

We do this as follows: as stated, we expand on the work of Topan et al. (2020). Therefore, the focus is specifically on the service part and generic control tower functionalities. We do this as parties in the supply chain express interest in managing their activities surrounding agreements on service delivery (i.e., service level agreements). However, setting up a CT environment is non-trivial; therefore, service functionality in such an environment adds extra complexity. We define dilemmas in setting up an SCT environment as design decisions in the system of interest. To conclude, we provide a reference architecture, which is usable for setting up specific system components oriented for usage in a maintenance setting.

**Table 1:** Summary of strengths and gaps in CT architectures

Paper	Focus	Strengths	Gaps
Shou-Wen et al. (2013)	Supply Chain Information Control Tower	Multi-layered architecture; Contains a wide range of technologies	Relations between components unclear; Does not describe IT functionality
Alias et al. (2014a)	Supply Chain Control Tower Architecture	Generic architecture; Continuous feedback loop	No specific domain focus; Not an IT architecture
Baumgrass et al. (2014)	Transportation Control Tower	Specific focus on transportation; Contains software components	Unclear how components fit business requirements
Hofman (2014)	Control Tower Architecture for Multi- and Synchromodal Logistics	Specific focus on logistics; Interfacing between components	Details on interfaces with other CTs are missing; Unclear which software should be used
Rustenburg (2016)	Control Tower ICT architecture for spare parts planning	Specific focus on spare parts; Maintenance-oriented	Microsoft heavy IT architecture; Exact configuration unknown
Liotine (2019)	Pharmaceutical Supply Chain Control Tower	Functional architecture for pharmaceutical logistics	Not an IT architecture
Topan et al. (2020)	Service Control Tower process architecture	Process architecture; Strong empirical foundation	Does not contain an IT architecture

## A reflection on the (service) control tower concept and a methodology to generalize it

In the previous section, we researched the literature on (S)CTs. However, an academic discussion on the purpose and originality of the (S)CT concept is necessary. We (partially) start this discussion in this paper by conceptualizing and generalizing the core principles behind a CT. In this section, we (critically) reflect on the (S)CT concept. We do this by looking at the available literature and IT concepts/systems with similar applications in supply chain or business activities. We then discuss the practical feasibility and originality by examining the business implications. We end this section by describing our methodology for generalizing the (S)CT and developing a specific reference architecture application.

However, as stated earlier, there is literature available that also adds to this discussion. The white paper by Bleda et al. (2014), from which the CT definition is often cited in academic literature, is one of the earliest publications. However, as stated, it is a white paper, which means that the firm authoring the paper (i.e., Accenture) has a commercial interest in selling the concept to its customers. One could see it as the next version of an IT system that optimizes supply chain activities. The unanswered question, therefore, is, is the CT concept a form of old ideas parading as new ones? A strength of the CT concept is that the physical CT, for aviation purposes, is used as a metaphor for the IT version. This metaphor often resonates well with companies and organizations interested in a CT system. However, the discussion then often steers into the field of application: what processes does the CT control?

**Fig. 1:**
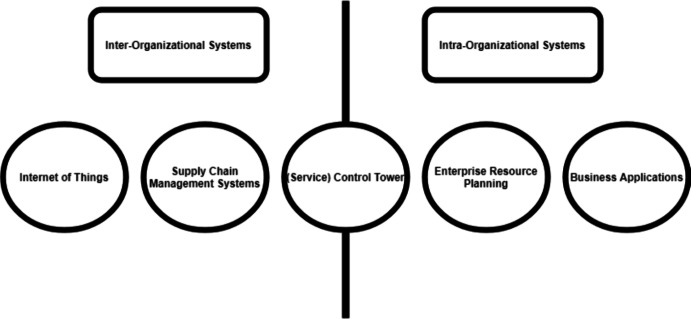
The (service) control tower positioned between intra- and inter-organizational systems

The (S)CT is an intra- and inter-organizational system; it can control and support the internal business processes for single organizations, such as managing inventory for retailers with multiple physical stores. However, it can also link suppliers to this inventory management system, converting it into an inter-organizational system and operating in a way that would typically classify as a Supply Chain Management System (SCMS). If multiple actual physical objects feed (real-time) information into the (S)CT, one could link it to the Internet of Things concept. We visualize the position of the (S)CT in Fig. 1.

**Fig. 2:**
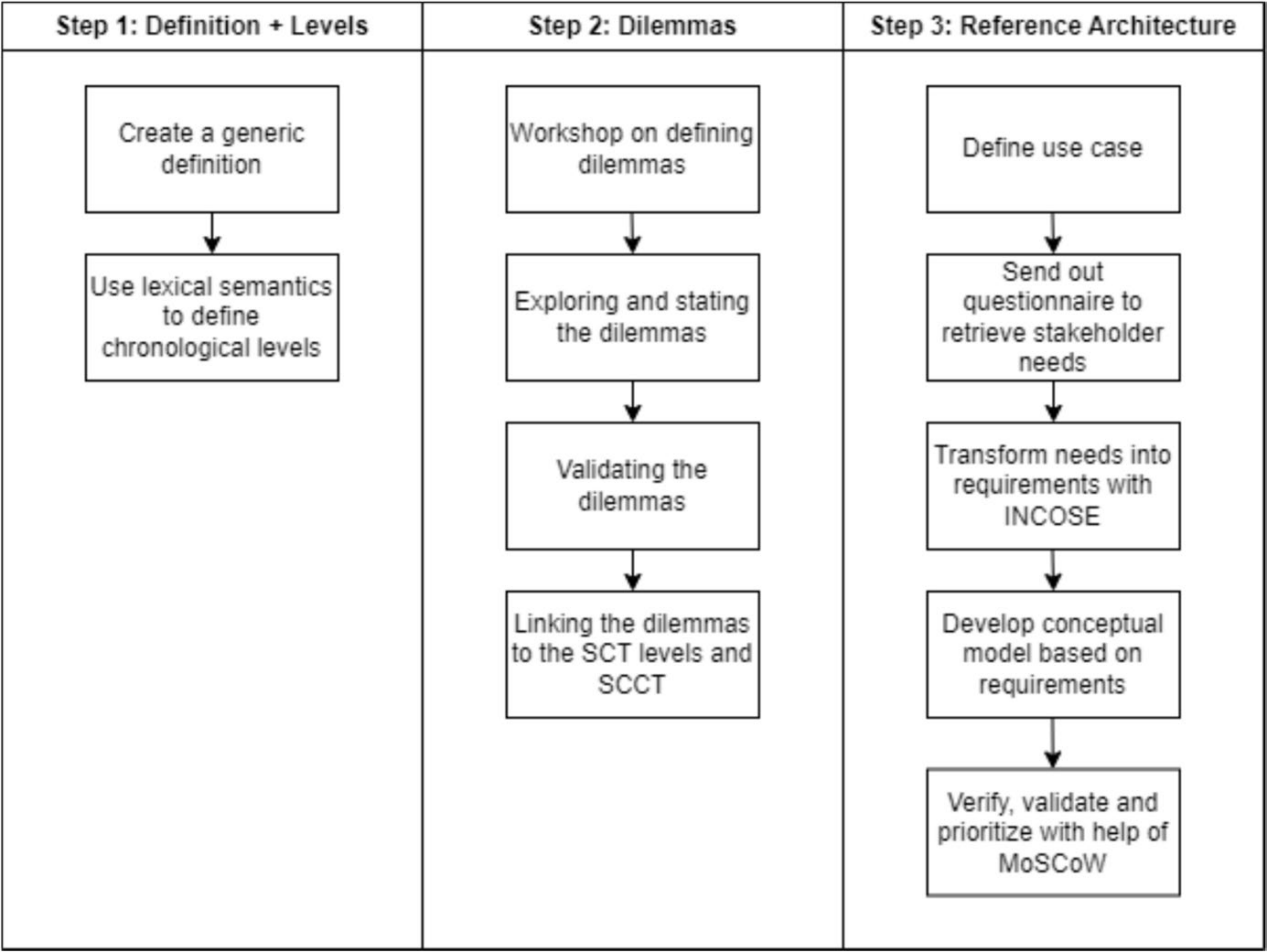
Methodology to define and develop a (S)CT

However, moving from an intra-organizational system to an inter-organizational system requires additional investment and involves horizontal and vertical collaboration in the supply chain. For vertical collaboration (i.e., organizations with the same business activities), the process of supply chain collaboration is hard, as the business model and value-added activities are similar, therefore, the incentive to collaborate is less natural and requires more strict coordination. The collaboration is logical for horizontal collaboration (e.g., between a retailer and its suppliers), as they already collaborate but often do not integrate their IT systems. An (S)CT could facilitate collaboration and act as an integrator of an IT system Fig. 2.

There is also a perceived difference between the business sector, collaboration between companies is more natural and embedded in their daily business operations (e.g., between product integrators and end users). In other sectors, competition is high, so collaboration is less incentivized by the urge to out-compete competitors. To set up this collaboration process, one needs to acknowledge the difficulties in this process and the choices that organizations need to make in development. The question still stands: Is the (S)CT necessary in this process, and if so, how can the construction of such a system be streamlined?

We approach this process of developing such a(n) (S)CT from multiple points of view. We start by (re-)defining the concept of the (S)CT, and, additionally, based on the combination of words (i.e., Service Control Tower), we apply lexical semantics to decompose individual words into so-called levels, which represent different chronological stages in the construction of an SCT environment. Next, we investigate the issues that might arise in the (inter-organizational) collaboration of setting up the SCT environment. We do this by phrasing dilemmas, topics related to the development of an SCT, and in which we position two alternative solutions on the extreme ends of the spectrum of possibilities. We derive these dilemmas based on a workshop and discussions with a group of expert stakeholders. In a separate session, we validate these dilemmas with the same group of people, after which we link them to different stages in the collaboration process and levels of the SCT.

We end this paper with a reference architecture for an SCT based on a use case in the maritime industry. We use this use case as an observational case study, as described by Wieringa (2014), in which the use case is a real-world phenomenon, but there is no direct intervention. However, we use analytical induction to generalize the problem posed in the use case and validate the reference architecture design.

There is an extensive academic debate about whether the single-use case is generalizable to a certain extent. Käss et al. (2024) describe the use of case study research in Information Systems research. They describe two types of single case studies, marginal and in-depth case studies, and the latter one needs to be critical, unusual, common, revelatory, or longitudinal. Our case study meets the unusual and longitudinal characteristics, which we will explain by introducing the use case. Next, we align with findings by Evers and Wu (2006), which state that a case study can provide certain generalizations as long as it adheres to the exploration of empirical knowledge and abduction. Easton (2010) argues that the single case study can provide general results if a critical realism perspective exists.

First, we introduce the use case and the stakeholders involved. Next, we send out a questionnaire to retrieve individual needs from stakeholders. We transform these needs into requirements with the help of Hull et al. (2011) and the INCOSE Guideline for Requirements (see INCOSE (2017)). We use the resulting requirements to categorize software elements as part of an SCT for a specific use case. We end with verifying and validating the reference architecture and, additionally, prioritize the development of particular software elements over others with the help of MoSCoW.

As stated earlier, the use case of interest is the maritime industry. The MAritime Remote CONtrol tower for service logistics Innovation (MARCONI) is a research project in which multiple maritime companies and public organizations collaborate to research and develop an SCT environment.^2^ The consortium comprises but is not limited to, vessel operators, system integrators, original equipment manufacturers (shipbuilders), and knowledge institutes (i.e., universities). The main goal is to facilitate and enhance the availability of high-value assets (i.e., ships and vessels) with the help of an SCT. However, multiple parties in the consortium are condemned to each other due to the limited competition in the maritime market in which they operate. Herefore, this influences how an SCT should operate to facilitate the processes of interest. Table 2 summarises the main consortium partners.

**Table 2:** Key stakeholders in the MARCONI consortium

Type of company	Number of employees	Main product/purpose	Expertise provided to the use case
Dredging company	11,000 ±	Dredging activities	Fleet management
Systems integrator	81,000 ±	Radar equipment	Life cycle services, service design, servitization
Original equipment manufacturer	12,500 ±	Vessels	Integrated logistics support
Original equipment manufacturer	3,000 ±	Vessels	Integrated logistics support
Naval force	11,000 ±	Security on sea	Service supply chain management
Vessel operator	1,000 ±	Providing maritime pilots	Fleet management
System integrator	550 ±	Integrating maritime systems	Service logistics

## The service control tower: levels and definition

An aviation CT is an exciting metaphor for the more IT-driven (S)CT. In Sections 2 and 3, we reflected upon the definitions from literature and the more commonly used definition from practice (i.e., Bleda et al. (2014)). We think the definitions are either too focused on a specific niche (e.g., Milenković et al. 2019) or are generic and ambiguous in the needed (technical) requirements (e.g., Alias et al. 2014b; Verma et al. 2020). We try to find a hybrid solution, incorporating the different options in the organizational scope, broadness of the supply chain, the direct availability of data and amount of servitization applied, mainly in line with the main objectives of a CT discussed by Trzuskawska-Grzesińska (2017). Therefore, we define the (service) control tower as ’an (inter-) organizational system which uses (real-time) IT to optimize the control of (a part of) the (service) logistics supply chain.’

**Fig. 3:**
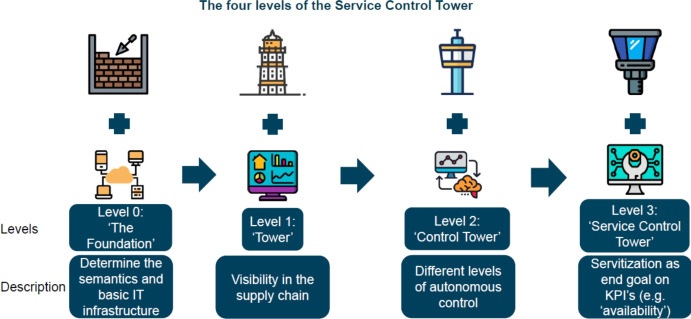
Four Levels of the service control tower

Based on our definition, we extract the meaning of service control tower mainly from the individual words composing the whole term. For example, we reflect on the meaning of the word ’tower’ or the combination of words (e.g., ’control tower’) in the context of the IT artefact it should represent. We, therefore, also see a link between the wording and the three phases Bhosle et al. (2011) describes creating visibility in a CT environment. Additionally, Liotine (2019) describes technology levels related to the storage, capture and accessibility of data. As shown in Fig. 3, we recognize four levels in developing a SCT artefact. First, as a foundation, organizations should create a basic semantic and IT infrastructure, enabling visibility in the supply chain with the help of dashboards. The next step initiates (autonomous) control on different aspects with, as an end goal, steering based on the servitization of Key Performance Indicators. For example, we discuss each level based on the MARCONI use case (i.e., supporting and maintaining (high-value) vessels).

### Level 0 - the foundation

Every artefact has a basis. For the (S)CT, the base is the foundation. At this level, organizations should determine the business architecture context, the available data, data capturing systems and the current (IT) systems. Combining these three triggers developing a common data standard and a blueprint for an IT architecture. These relate to the notion by Liotine (2019) that basic technology requirements should be in place regarding data exchange.

The foundation helps to set and define the semantics (i.e., data standards) and IT systems that must align to exchange data for the (S)CT environment. Data standards that organizations could consider in the maritime sector are wide; we give a few, but the options are broad. An example of standards are the One Maritime Data Standard,^3^ S1000D^4^ and EDIMAR.^5^ Which IT systems to align depends highly on the types of systems available and the desired integration level. This alignment could be done on an ERP level basis (i.e., linking existing ERP systems with the (S)CT) or more on an asset basis (i.e., linking systems from the vessels, e.g., AIS or sensors from the ship, directly to the (S)CT).

### Level 1 - the tower

At this level, the (S)CT can give users visibility and insight into the performance of the accompanying business processes. Therefore, data transformation and cleaning activities show the current status of the business processes. Organizations develop dashboards for monitoring and alerts, including selecting crucial Key Performance Indicators. Additionally, these dashboards provide support on a day-to-day basis in operations. This level complements the different levels of visibility as described by Bhosle et al. (2011).

For example, in the maritime industry, such a dashboard could give an overview of the current status of the vessels and the maintenance and other logistics activities that support their operation. At the tower level, such a system should show the current issues and alert the user of potential problems, but it should not have any direct influence or control over these processes.

### Level 2 - the control tower

Organizations can control the processes in the system based on the visibility of the supply chain. At this level, organizations need to determine which level of control and interventions are desirable in a certain process. Control in this sense also means that a specific target state should be achieved (e.g., on availability of the asset^6^) by applying the control tower and that the system can impose this in the supply chain. Based on these, a governance structure and different rules for decision-making should be put in place and implemented. As explained by Topan et al. (2020), exception messages could be automated while interventions can stay manual. Additionally, different levels of automation, but also different strategies, can be applied (e.g., Hofman 2014, Rustenburg 2016).

For definitions of levels of automation, see, for instance, Vagia et al. (2016). Different levels of automation are possible, and the system could operate in the most extreme variant, independent of human interaction. In the case of the maritime industry, there could be a hybrid of options. Specific actions in the (S)CT could be taken without human interaction (e.g., automatic procurement of spare parts and computing the optimal obsolescence strategy). Human confirmation is necessary for other events (e.g., replanning maintenance activities and assigning personnel to specific activities).

### Level 3 - the service control tower

The final level enables the system to optimize the supply chain based on servitization, which is ’the transformational process of shifting from a product-centric business model and logic to a service-centric approach.’ (Kowalkowski et al. 2017). In other words, companies shift from selling a product to selling a complete package, which includes service-related activities. We envision the SCT focusing more on servitization principles (i.e., integrating products and services, tailoring the outcomes to customer needs and a recurring revenue model).^7^ In other words, the artefact becomes a full-fledged service control tower. Contrary to Verma et al. (2020), we think that control in the system based upon SLAs takes place in the last level. Organizations must decide which processes are eligible for service contracts, implement them in the system, and set up a legal basis. The SCT will evolve into a service-oriented architecture, complementing the process architecture by Topan et al. (2020).

In the case of keeping maritime assets up and running, organizations owning these assets usually have SLAs in place, explicitly stating which kind of service an asset owner can expect for certain parts of the vessel. These SLAs typically state a specific lead time, service level or a combination of both for which a supplier delivers a particular service. An SCT should support the monitoring and execution of the performance indicators in an SLA. However, asset owners are more interested in the bigger picture of keeping the asset running. A more advanced version of a CT, i.e., an SCT, will focus on KPIs like the availability of an asset, the amount of downtime or the costs of upkeep.

## The service control tower: dilemmas

When collaborating in a supply-chain context, companies’ strategies can lack synergy in certain aspects. In other words, their (business) interests might diverge on particular topics. Therefore, in setting up an SCT, organizations face development dilemmas. For example, in setting up an SCT, how is governance of the system and control taking place (c.f., Chandra and van Hillegersberg (2018), Didden et al. (2021))? Setting up an SCT is collaborative, but the resulting system does not need to be.

We investigate these dilemmas by organizing workshops with companies that want to construct an SCT in a maritime maintenance setting (i.e., the MARCONI project^8^). Based on brainstorms, consortium partners introduced potential issues in setting up the environment. These were structured and then presented in an additional workshop where experts could review them, add final dilemmas and vote on a preferred direction per dilemma (i.e., preferring an alternative over another).

We phrase a decision on a specific topic as a dilemma (e.g., on ownership of an SCT environment). Next, we present two alternatives and extremes in the dilemma’s solution (e.g., centralized vs decentralized ownership). For organizations collaborating, these dilemmas are guidelines for making essential decisions on setting up a SCT environment and discussing these dilemmas at different stages in the development of an SCT (i.e., at the different SCT levels^9^ and the various steps in collaboration^10^). We position the alternatives in the dilemmas as two opposing solutions, but in reality, mixtures are possible (e.g., hybrid ownership). These are not best-practice solutions by definition but should stimulate a discussion between organizations to formulate and shape the most fitting solution for the SCT environment in development. Dilemmas offer an opportunity to cooperate and guard ethical norms, see Scalet (2006).

### The supply chain collaboration tool

Collaboration, especially in supply chains, is complex. However, there is experience with different forms of collaboration. For horizontal collaboration (i.e., between companies/organizations with similar business activities) in supply chains, the interests between parties are conflicting, as they often offer similar products or services. Vertical collaboration (e.g., between a supplier and retailer) is more straightforward, as the organizations frequently collaborate naturally. However, even for vertical collaboration, strategic business-related hurdles limit organizations’ willingness to collaborate more intensely.

**Fig. 4:**
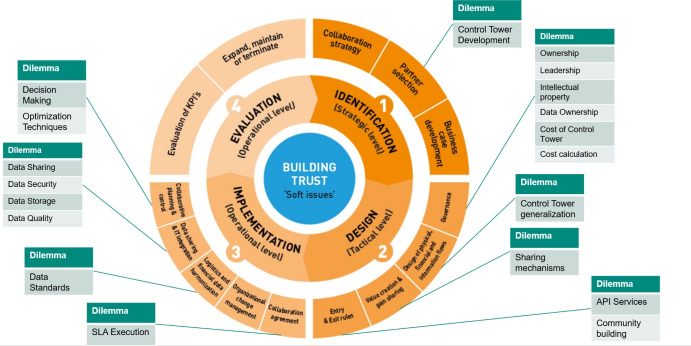
Dilemmas related to the Supply Chain Collaboration Tool

In the Netherlands, the Dutch Organization for Advanced Logistics, TKI Dinalog, has experience with a broad spectrum of collaborations in supply chains due to the public research they finance. As a result, they developed a Supply Chain Collaboration Tool that summarizes the gained experience based on difficulties in collaborating. It helps stakeholders in inter-organizational collaboration to structure the discussion. Next, specific discussions have overlapping themes (e.g., collaboration strategy and partner selection are both strategic), so grouping them together is valuable. Before constructing a system or collaboration intensives, the steps of the Supply Chain Collaboration Tool should be followed chronologically. The Supply Chain Collaboration Tool (seen in Fig. 4) describes four phases of collaboration.

In the first phase, identification, organizations must define and identify why they want to collaborate by building a business case and developing a collaboration strategy. The second phase, design, touches upon aspects like governance, the design of the financial and information flows, value creation, and gain sharing. For the third phase, the focus shifts from tactical design to operational implementation. Organizations must cover topics like harmonization of data, integration of IT and data sharing and change management processes. In the final and last phase, organizations evaluate the KPIs underlying the collaboration and decide to expand, maintain or terminate.

Developing an SCT can be an intensive collaboration process in supply chains. Therefore, we link the Supply Chain Collaboration Tool to the dilemmas we extract from the organizations. The Tool helps recognize the chronological order of particular dilemmas in creating an SCT and recognizes distinctions on strategic, tactical, and operational levels. Additionally, the different SCT levels, as defined in Section 4, could act as the different iterations of the Tool.

### Control tower dilemmas

We extract two types of dilemmas based on workshops and discussions in the MARCONI project. The first type is called technical dilemmas, which we define as dilemmas regarding design decisions in the hardware or software of the system (e.g., on the programming language, data storage or data governance). The second type is business dilemmas, which we define as dilemmas encountered in the business context surrounding the artefact of interest (e.g., intellectual property ownership).

In total, we identified 19 dilemmas. Six of these are business dilemmas, eight are technical dilemmas, and the remaining five are a combination of technical and business dilemmas. For all dilemmas, we identify options A and B. One could see these as radical alternatives to one another. However, the solution does not need to be exclusive to either side.

Next, we link these dilemmas to the Supply Chain Collaboration tool and the different levels of the SCT. We do this by aligning the definition of the dilemma with the component in which the activities logically align. Most business-related dilemmas occur early in the collaboration, while technical dilemmas occur later in the cooperation. Additionally, most dilemmas (thirteen) must be discussed early in constructing the SCT, i.e., at level zero, while a few only occur at later levels. Finally, we summarize the dilemmas, their options, classification and relation to the tool and the SCT levels in Tables 3 & 4.

**Table 3:** Overview of SCT dilemmas: Options

Dilemma	Option A	Option B
Control Tower Development	In-house	Outsourced
Ownership	Centralized	Decentralized
Leadership	Lead company in consortium	Independent
Intellectual property	Shared	Partially owned
Data Ownership	Single organization	Distributed
Cost of Control Tower	Transaction based	Subscription based
Cost calculation	Transparent	Black Box (Individually determined)
Control Tower generalization	Generic	Tailor-made
Sharing mechanisms	Legal determined	Algorithmic determined
API Service Access	Open API	Closed API
Community Building	Open	Closed
SLA Execution/Collaboration	Rule-based (Smart Contracts)	Trust-based
Data Standards	Single	Multiple
Data Sharing/Gathering	Real-time	On-demand
Data Security	Encryption only	Blockchain
Data Storage	Centralized	Decentralized/Distributed
Data Quality	Cleaned data	Raw data
Decision Making	Centralized	Decentralized
Optimization Techniques	Exact (OR)	Self-Learning (ML)

**Table 4:** Overview of SCT dilemmas: Details

Dilemma	Classification	Step in collaboration tool	SCT Level necessary
Control Tower Development	Technical + Business	Partner Selection	0
Ownership	Business	Governance	0
Leadership	Business	Governance	0
Intellectual property	Business	Governance	0
Data Ownership	Technical + Business	Governance	0
Cost of Control Tower	Business	Governance	0
Cost calculation	Business	Governance	0
Control Tower generalization	Technical	Design of physical, finance and information flows	0
Sharing mechanisms	Technical + Business	Value creation and gain sharing	2
API Service Access	Technical	Entry & exit rules	1
Community Building	Business	Entry & exit rules	0
SLA Execution/Collaboration	Technical + Business	Collaboration agreement	3
Data Standards	Technical	Logistics and financial data harmonisation	0
Data Sharing/Gathering	Technical	Datasharing & IT integration	0
Data Security	Technical	Datasharing & IT integration	0
Data Storage	Technical	Datasharing & IT integration	0
Data Quality	Technical	Datasharing & IT integration	1
Decision Making	Technical + Business	Collaborative planning & control	2
Optimization Techniques	Technical	Collaborative planning & control	2

In Fig. 4, we look more in-depth at the dilemmas compared to the Supply Chain Collaboration Tool. Here, we see two clusters that contain the most dilemmas. There, we see many (business) dilemmas in the governance step of the collaboration tool. As an example, dilemmas that supply chain parties should discuss are related to ownership (centralized or decentralized), leadership (single lead organization versus a consortium) or the computation of costs (transparent or black box). Another cluster of a mainly technical-oriented step is within the collaboration tool’s data sharing and IT integration. In this step, organizations should discuss different aspects of data, i.e., data sharing/gathering, security, storage and quality. In the other steps, fewer dilemmas are observable, such that organizations should focus extra on the governance and, data sharing and IT integration steps in collaboration. However, that does not mean the other dilemmas are useless in an SCT design.

**Fig. 5:**
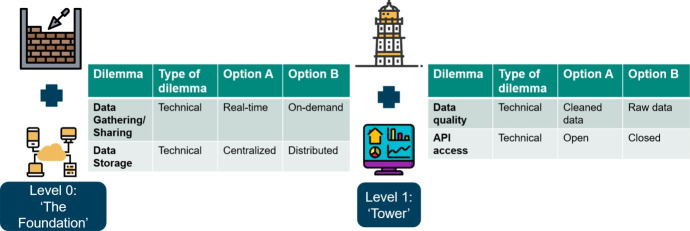
Dilemmas in the first two SCT levels

If we put the dilemmas in perspective towards the different levels of the SCT that we defined, most dilemmas are related to level zero. For example, Fig. 5 shows a few dilemmas linked to the first two levels, that of the foundation and the tower. At level 0, the foundation level, an example of dilemmas encountered are data gathering/sharing and data storage, which relate to the data sharing and IT integration step in the Supply Chain Collaboration Tool. Level 1, the tower level, contains two dilemmas, and the first discusses the Application Programming Interface Service Access. In other words, are there possibilities for other parties to access data in the system, or is the SCT environment generally closed? Additionally, the parties involved need to decide whether cleaned data should be the standard or raw data in the visibility step is allowed.

**Fig. 6:**
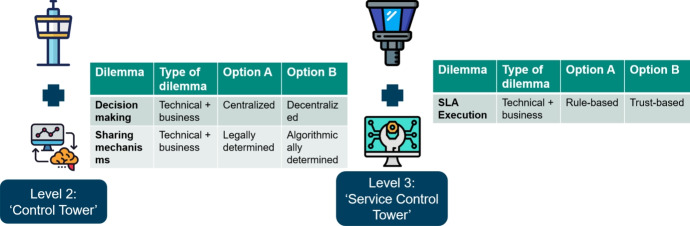
Dilemmas in the last two SCT levels

For levels 2 and 3, we recognize three dilemmas related to level 2, the control tower level and only one to level 3, the service control tower (see Fig. 6 for a section of those). At the control tower level, parties involved should discuss the decision-making and whether this should be centralized or decentralized. Additionally, sharing mechanisms could be legally determined or done by an algorithm (e.g., based on cooperative game theory concepts). The service control tower level focuses mainly on the inclusion of SLAs. Therefore, organizations should discuss whether the execution of these SLAs should be rule-based within the system or done in a more lenient trust-based matter.

We validate the seven most essential dilemmas by letting nine stakeholders vote^11^ for their favourite option. We visualize the results in Table 5. In this table, it is visible that the stakeholders are not unanimous on one of the dilemmas. This non-unanimity indicates that the dilemmas are discussion points for which no clear best practice solution exists. It also shows that in the use case of interest, the actual realization of an SCT is still far off.

**Table 5:** Voting for favourite Control Tower Dilemma options

Control Tower Dilemma	Option A	Option B
Decision Making	Centralized (37.5%)	Decentralized (62.5%)
Ownership	Centralized (37.5%)	Decentralized (62.5%)
Optimization Techniques	Exact (Operations Research) (44.4%)	Self-Learning (Machine Learning, AI) (55.6%)
Data Sharing/Gathering	Real-time (44.4%)	On-demand (55.6%)
Control Tower Development	In-house (66.7%)	Outsourced (33.3%)
SLA Execution	Rule-based (Smart Contracts) (77.8%)	Trust-based (22.2%)
Control Tower generalization	Generic solution (22.2%)	Tailormade system (77.8%)

To conclude this section, organizations could benefit from our identified dilemmas, offering them structure and creating a discussion space. These dilemmas are flexible, so they might change over time, i.e., if technological or economic advancements create new dilemmas or erase current ones. Our early investigation could help researchers and organizations involved in supply chain collaborations use our dilemmas as a basis for more empirical research. Unfortunately, little knowledge is available about which decisions in the design of an SCT should be best-practice and how they relate to a certain context (i.e., consortium of partners). However, the dilemmas could be a valuable framework for developing best-practice solutions.

## The service control tower: reference architecture for the maintenance of (high-value) assets

We design a reference architecture to maintain (high-value) assets for an SCT environment that adds to the dilemmas. In other words, we develop an SCT in abstract (software functionality) terms for usage in maintenance processes. This abstract focus is in line with Bass et al. (2003), who define a reference architecture as a ’reference model mapped onto software elements (that cooperatively implement the functionality defined in the reference model) and the data flows between them’. A reference architecture is usually a collection of systems that helps create a new system version (Muller 2008). Compared to the types of reference architectures defined by Angelov et al. (2009), we aim to develop a Type 5 reference architecture, i.e., an architecture that is for an immature innovative technology containing generic components. Next, our architecture links with the concept of Service Oriented Architecture, which links service offerings in a business context contrasting to more traditional ways of doing business (Erradi et al. 2006).

We develop the reference architecture based on input from the MARCONI project research consortium. To start, we define the context of the SCT. Next, we derive needs and inputs from potential users of the SCT in the maintenance setting. We transform the needs into requirements and check the requirements on quality and reference. The requirements then are used and mapped towards software components and their functionality. The resulting components fit in a reference architecture, which we base on the Enterprise Architecture principles, modelled with Archi^12^ according to the TOGAF (Josey 2016) methodology. Enterprise Architecture is"a coherent whole of principles, methods, and models that are used in the design and realisation of an enterprise’s organisational structure, business processes, information systems, and infrastructure.” Lankhorst (2009). TOGAF (The Open Group Architecture Framework) is a methodology for developing an IT enterprise architecture.

**Fig. 7:**
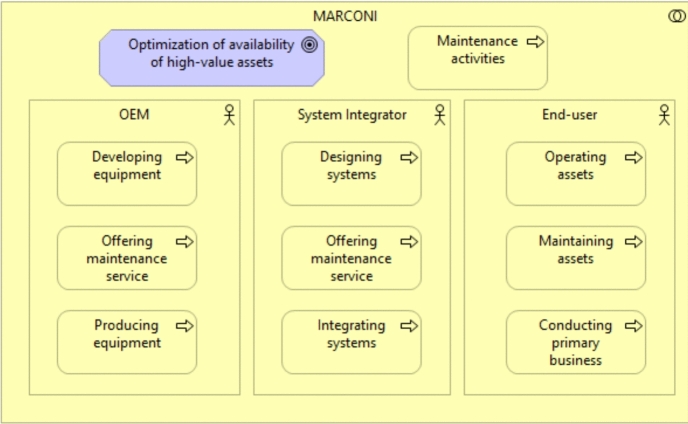
Business Context MARCONI Consortium

Within the MARCONI project, original equipment manufacturers (OEMs), system integrators, and end-users of high-value assets (i.e., ships and vessels) are active. We display them in Fig. 7. For an OEM, the main purpose is to develop and produce equipment and offer an additional maintenance service. A system integrator designs and integrates systems and offers maintenance for the upkeep. Last but not least, the end-user operates and maintains the assets for use in primary business. The end goal for the SCT is to optimize the availability of the (high-value) assets, while on the other hand, the SCT reduces costs for maintenance activities, where possible.

From workshops and a questionnaire, we derived 47 needs from this business context^13^. However, some needs contain multiple requirements. Therefore, we transform the needs and check them with the support of the guidelines of (Hull et al. 2011). When checking and transforming the needs into requirements^14^, we extracted 93 requirements. Nevertheless, only 80 requirements were deemed suitable^15^ for our architecture.

**Fig. 8:**
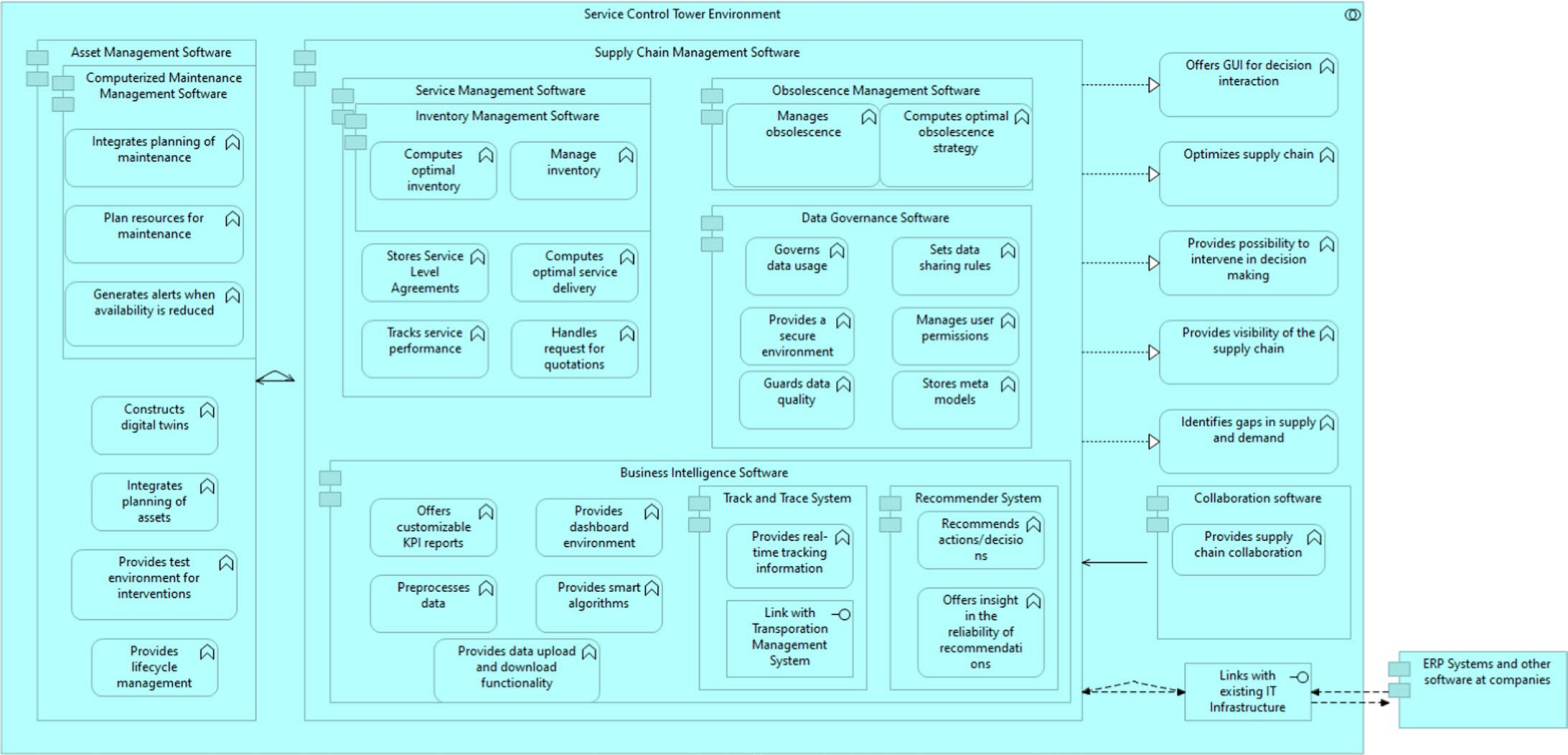
Service control tower Reference Architecture

We map these requirements towards software components in our architecture. In other words, we only describe the abstract system, the software components and their individual and combined functionality. We show the architecture in Fig. 8. We discuss the individual components and their functionality in more depth.

From discussions with potential users, an observation is that some aspects of the environment are less necessary than others. Therefore, we organized an additional MoSCoW (Must Have, Should Have, Could Have, Would Have) workshop^16^ in which we prioritized individual software components of the SCT. This workshop discusses the need for certain software components relative to others. The experts part of the workshop are either involved in developing an SCT environment or have years of experience in similar systems. We classify the software elements based on the plurality vote, in case of a draw, the experts will select their favourite between two alternatives. We discuss each software component based on its priority. No software elements are Would Haves.

### Must have

As a Must Have, we recognize two software elements. Business Intelligence is the first, defined as ’techniques used in spotting, digging-out, and analyzing business data, such as sales revenue by-products and/or departments, or by associated costs and incomes’ (Elena 2011). Experts in the workshop state that Business Intelligence software is at the core of a(n) (S)CT, as it visualizes the current state of the supply chain, and without it, a(n) (S)CT would lack any meaningful data to act upon. Data Governance is the second one, and we define this as ’the framework for decision rights and accountabilities to encourage desirable behavior in the use of data’ (Weber et al. 2009). Participants in the workshop stated that Data Governance is a pre-requisite in a(n) (S)CT between organizations. Organizations need to determine rules for sharing and governing data and, through time, alter and adapt. We see that Business Intelligence software can be linked to the visibility level of the SCT, while the Data Governance element is linkable to the foundation level.

### Should have

The SCT environment has a collection of Should Haves. These are Collaboration Software, Asset Management Software, Maintenance Management Software and a Track and Trace System. Collaboration Software facilitates supply chain collaboration processes (e.g., via video/audio or sharing files). We define Asset Management Software with the help of the ’systematic process of maintaining, operating and upgrading physical assets cost-effectively’ (McElroy 1999). Maintenance Management Software is a part of Asset Management Software and focuses on maintenance processes. At last, the Track and Trace system provides (real-time) tracking information on specific aspects of the supply chain (e.g., transportation, the current status of orders, and business events). The Track and Trace system would likely link to a Transportation Management System (TMS), to show the current status of transporting activities, when outsourced to third-parties.

### Could have

Two software components are categorized as a could have. Service Management Software supports and stores information regarding service delivery and offerings in the supply chain. This software supports the execution of the Service Level Agreements. The other element is the Recommender System Software. We define the Recommender System as ’any system that produces individualized recommendations as output or has the effect of guiding the user in a personalized way to interesting or useful objects in a large space of possible options.’ (Burke 2002). A Recommender System would help in searching for preferable actions/recommendations.

Users and organizations developing an SCT should look critically at which elements they need and which are less desirable. Next, our components focus heavily on the maintenance aspects and generic functionality. Therefore, in other contexts, other elements might be more suitable. Additionally, we lack research into the interfacing between the components and the data interoperability of these elements. However, three participants of the MARCONI consortium (i.e., an original equipment manufacturer, naval force and systems integrator) granted insights into the functionality of their IT systems, which we show in Table 6.

**Table 6:** Available IT systems related to SCT functionality at project participants

	Original equipment manufacturer	Naval force	Systems integrator
Asset Management Software	Enterprise Resource Planning	Enterprise Resource Planning	Enterprise Resource Planning
Computerized Maintenance Management Software	Fleet Management System	Enterprise Resource Planning	Enterprise Resource Planning
Service Management Software	Enterprise Resource Planning		Customer Portal Enterprise Resource Planning
Inventory Management Software	Fleet Management System Enterprise Resource Planning	Enterprise Resource Planning	Inventory Management Software Enterprise Resource Planning
Track and Trace System			Enterprise Resource Planning

The functionality that supports a lot of the core functions of the SCT reference architecture, especially those related to the maintenance activities. We have mapped these in Table 6, showing that Enterprise Resource Planning software is often the core IT system for these companies, used in different forms. However, the organizations involved in the research state that sometimes functionality is limited compared to an SCT. For example, organizations in the MARCONI project mention that maintenance software is available but is nowhere near predictive in its current form. The most striking feature of the available IT systems is the missing interfaces (i.e., data exchange) with other organizations. Therefore, we see the SCT for maintenance of (high-value) assets as an inter-organizational system of existing IT systems, which are linked with additional (more advanced) functionality, most importantly, Business Intelligence and Data Governance to streamline the linking of these systems and extract supply chain information. In the end, the system should predict in a more advanced manner when maintenance is necessary.

**Fig. 9:**
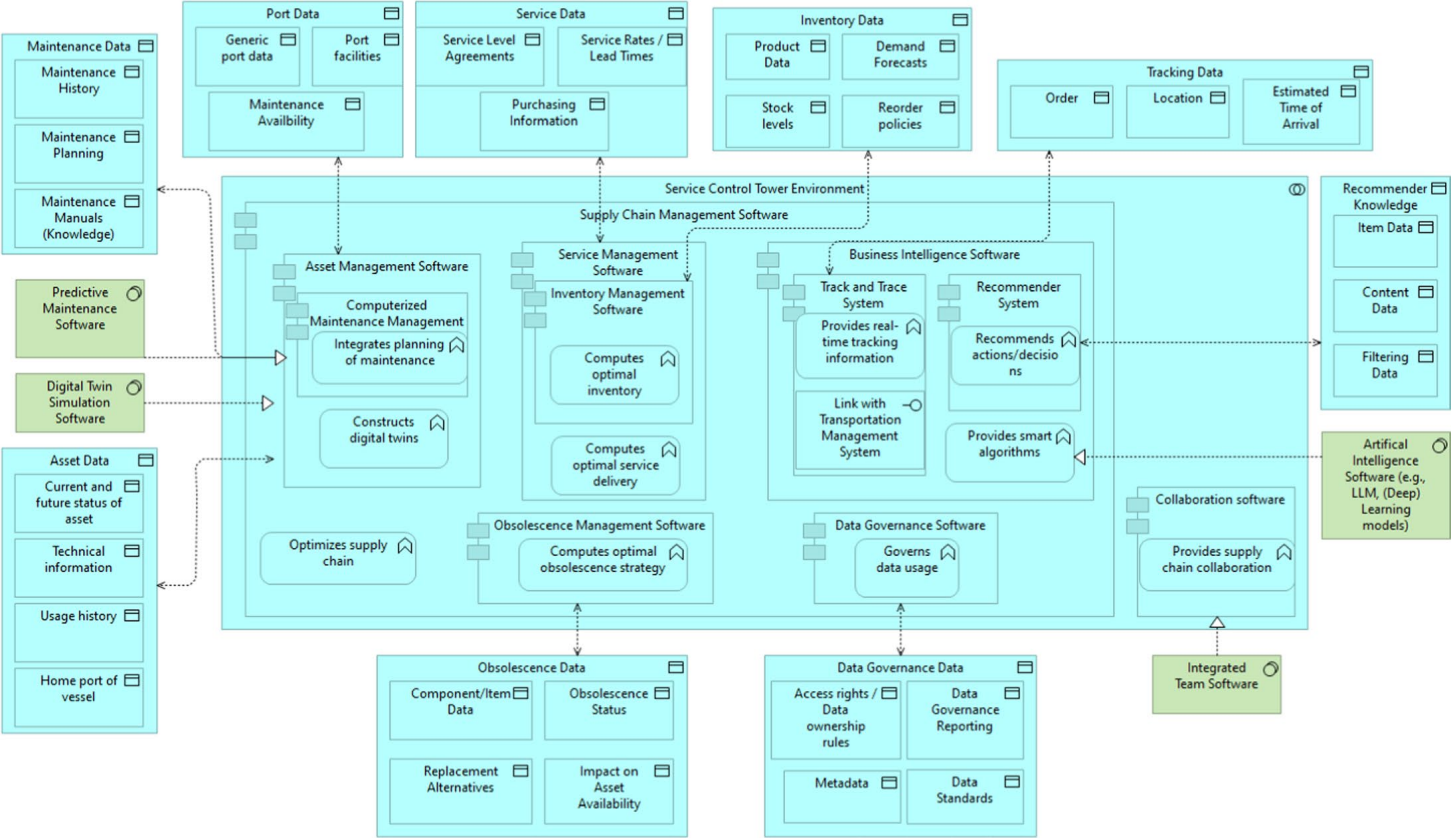
Service Control Tower Data Architecture

To prepare for such a system, we sketched the first data architecture for the SCT reference architecture. In this model, we show, on a generic level, which data objects and system software to expect for the SCT of interest. We map these to the individual applications and focus on single functionalities per application. We selected the latter based on its impact on an advanced version of the SCT. The Data Architecture is visible in Fig. 9.

The question is, how could such a system operate in maritime service logistics? Assume we have an asset, in this case, a dredging ship, operated by a dredging company. The ship most likely operates worldwide and sends data on the current status via radio signals or satellite connection (e.g., the status of dredging, operational time, etc.). The company operating the vessel has technical information on the vessel but also knows the history of usage. This data, which can contain, for example, fuel usage, rotations per minute, failure rates, and repair rates, is of interest to the maintenance planning of the SCT. In combination with the digital twin simulation and predictive maintenance software, the system tries to plan future maintenance, which incorporates the availability of ports (through port data) to allow for dry-docking,^17^ if necessary, and align this with future usage schedules. During maintenance, spare parts must be present, which helps in servicing the vessel. The service management software communicates with the asset management software on the expected maintenance activities and looks for the current inventories and service level agreements. Based on this, it assists in purchasing the spare parts, which aligns with the service level agreements in place.

However, if specific parts are not available anymore, i.e., a part is obsolete, either from a technical or functional perspective. In that case, the obsolescence management software helps, it looks, with help from the recommender system, for an alternative spare part, which could keep the asset functional as expected. If such replacements are available and ordered in the system, the track and trace system keeps track of the estimated time of arrival and order status. The main goal for this part of the SCT is to manage the supply chain so that the availability of the asset is as high as possible.

More generic functionality is in the data governance software. Here, users can have limited access to certain data. For example, a pilot of a specific ship only has access to information on that ship, not other ships in the fleet. For maintenance managers, the perspective is different; they have access to maintenance-related data but are likely unable to see sensitive data on the operational status of the vessel. In the case of inter-organizational collaboration, integrated team software could easily facilitate communication between organizations (e.g., a systems integrator and end-user) on the supply chain status. With intelligent algorithms (e.g., LLMs, deep learning models, etc.), the after-sales service logistics can be optimized further, improving overall supply chain efficiency.

**Fig. 10:**
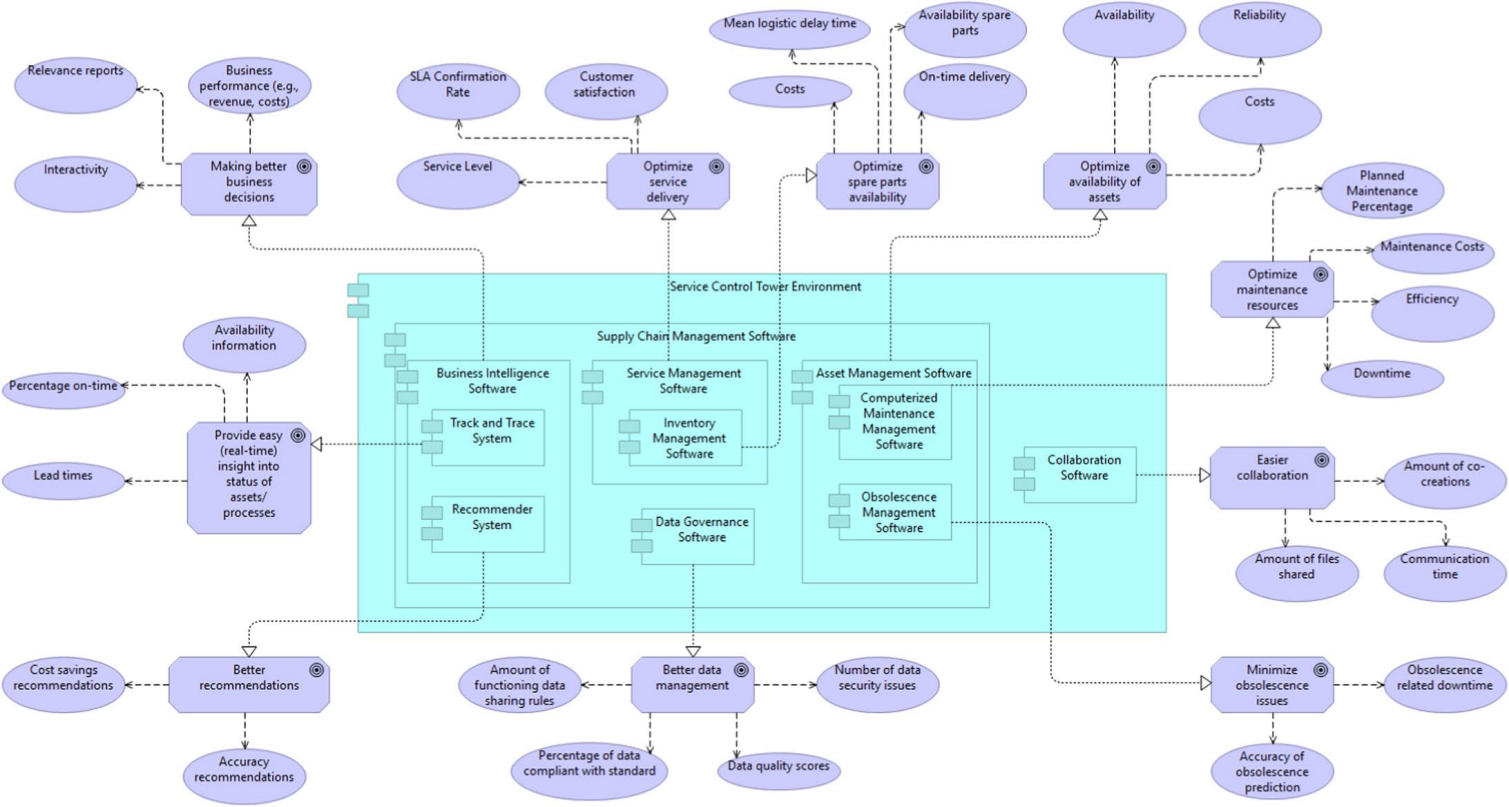
Service Control Tower Performance Measurements

How could this performance gain be measured in an SCT in the end? Fig. 10 shows the individual goals per software module and performance measurements. For example, for a vessel connected to the SCT, the asset management software should optimize the availability (e.g., percentage of time available) and reliability and consider the costs for availability. The maintenance for this vessel should be optimized, focusing on balancing the maintenance costs, efficiency and downtime of the vessel itself. The other software modules are also described in Fig. 10, but alternative performance measurements are also possible as long as they contribute to the goal of the individual module.

## Conclusion and discussion

This paper explores the CT as an IT system that optimizes a (part of a) supply chain. The concept is in line with the traditional view of the inter-organizational system. However, CTs’ usage depends on the context; some are in a specific field (e.g., transportation or spare parts), while others are generic. Therefore, this paper focuses on a particular case of CT, namely the SCT and how to initiate collaboration between supply chain partners. We show that collaboration takes place in four phases. Additionally, we identify four levels of the SCT that partners need to construct to create a complete SCT.

Additionally, we identify nineteen dilemmas in the development of an SCT. The dilemmas are either business dilemmas, technical dilemmas, or both. We link the dilemmas to the different phases of collaboration and levels of the SCT. Most dilemmas occur early in the design phase and at the foundation level of the SCT. Special attention is needed to SCT governance, data sharing, and IT integration. Finally, we provide SCT designers with different alternatives for setting up the system.

Last but not least, we design a reference architecture for an SCT in maritime maintenance. First, we create the system as a software system with different individual but interacting components based on expert knowledge. Next, we rank the individual components based on their priority. We provide a data architecture and performance measurement architecture to add to this reference architecture. Potential designers and future users of such a system can use our architectures as the first blueprint for their specific solution.

In this paper, we face multiple limitations. First, the recognized collaboration and SCT construction dilemmas are merely exploratory research. Empirical evidence is required to assess which dilemmas are more persistent. Second, we have approached these dilemmas from a maritime maintenance context, and other dilemmas might occur when a different business context is at play. Third, our provided reference architecture is on an abstract level. Finally, organizations require more details to set up control towers (e.g., on data standards, interfacing, and the functionality of individual processes and components). At last, research should determine which SCT configurations are inherently more successful than others.

## Appendix 1: Raw system needs MARCONI control tower

In the consortium we conducted a questionnaire and also had multiple plenary (and Skype) sessions. Based on this, we defined needs. We phrase them in the form of ’MARCONI should’. We give the list here below, which is unsorted based on the needs gathered in the questionnaire and different sessions.

- MARCONI should optimize the supply chain planning at tactical and operational level
- MARCONI should enhance visibility and the links in the supply chain network
- MARCONI should offer the possibility for a supply chain collaboration (NVP).
- MARCONI should offer operators the possibility to intervene in short-term decision making with predefined rules
- MARCONI should measure service contract performance
- MARCONI should offer the possibility for KPI reports
- MARCONI should offer the possibility to add KPI’s
- MARCONI should provide real-time supply data
- MARCONI should provide real-time demand information
- MARCONI should identify gaps in demand and supply
- MARCONI should generate alerts to prevent reduction in availability
- MARCONI should provide a test environment for reactive and proactive interventions on short term system performance
- MARCONI should offer the possibility of integrated planning
- MARCONI should offer the possibility of obsolescence management
- MARCONI should offer a secure environment
- MARCONI should offer a connection to internal IT systems/infrastructure of participants
- MARCONI should offer a recommender system
- MARCONI should offer insight in reliability of the recommendations
- MARCONI should offer possible actions to follow up on the recommendations
- MARCONI should offer a dashboarding environment (Business Intelligence)
- MARCONI should offer the possibility for life cycle management
- MARCONI should give insight in fuel and emission metrics
- MARCONI should give the possibility to go from a stovepipe system to a more cross-functional system
- MARCONI should provide an overview of people and processes involved in the service logistics in company
- MARCONI should provide an end-to-end overview of the service logistics supply chain
- MARCONI should align logistics processes between supplier and customer
- MARCONI should give an indication of lead time on (spare) parts
- MARCONI should give information about possible suppliers of (spare) parts
- MARCONI should give information about the repair or replace process of parts of systems
- MARCONI should give information on preventive or corrective maintenance taking place
- MARCONI should give information about the potential cause of failure in case of corrective maintenance
- MARCONI should provide optimized maintenance plans including specific tasks and indication on resources needed
- MARCONI should provide decision support models (algorithms)
- MARCONI should have adaptable algorithms for optimization
- MARCONI should data download and upload functionality
- MARCONI should be able provide a GUI for system interaction with planning models
- MARCONI should support a network of organizations
- MARCONI should contain to option to construct a digital twin
- MARCONI should support machine learning algorithms
- MARCONI should reduce planning workload
- MARCONI should achieve reduction of disruption of primary activities
- MARCONI should provide a track and trace functionality
- MARCONI should give the asset-owner an overview of the core operations
- MARCONI should in real-time show the operations of the assets
- MARCONI should facilitate the addition of sensors on assets linked to the control tower
- MARCONI should offer the possibility to actually control equipment
- MARCONI should offer the owner of the asset to use pluggable intelligent tools or not

## Appendix 3: Service Control Tower MoSCoW results

Here we visualize the results of the MoSCoW workshop. In case of a draw, the authors of this work had the definitive vote on classification. We weighted votes equally between participants. A bold number means most votes of experts went to a certain classification of MoSCoW, or, in the case of collaboration environment, the discussion after voting resulted in preferring should have over must have.

**Table:**

Software Element	Must Have	Should Have	Could Have	Would Have
Collaboration Environment	3	**3**	2	0
Data Governance	**7**	1	0	0
Service Management	1	2	**4**	0
Business Intelligence	**5**	3	0	0
Track and Trace	2	**6**	0	0
Recommender System	1	2	**5**	0
Asset Management	2	**4**	1	1
Maintenance Management	3	**4**	1	0

## Footnotes

1 TKI Dinalog is a knowledge consortium that funds public-private cooperation in the Dutch logistics sector. Next, the organization supports valorization activities and provides R&D support.

2 See the following links for more in-depth information. Project information from NWO and project information from TKI Dinalog.

3 An initiative between Boskalis, Damen, IHC and Van Oord.

4 Part of the S-Series of Integrated Logistics Support Series.

5 Electronic Data Interchange for the European Maritime Industry.

6 We focus on the goals later on in this paper.

7 We emphasize that this does not imply that ownership of an asset is for the party that offers the servitization, as is often the case with more strict servitization.

8 MARCONI stands for: Maritime Remote Control Tower for Service Logistics Innovation and is a Dinalog/NWO funded project that tries to optimize the availability and usage of resources of maritime assets. The project contains a consortium of universities and maritime companies. For more info, see this website. See Section 3 for additional information.

9 See Section 4.

10 See Section 5.1.

11 Voting is not mandatory, but voluntary, which results in two dilemmas (i.e., decision making and ownership) not having nine but eight stakeholders voting for it.

12 See https://www.archimatetool.com/.

13 See Appendix 1.

14 See Appendix 2.

15 We checked the requirements on validity and correctness with the help of Hull et al. (2011) for requirements.

16 See Appendix 3.

17 These ports, should therefore, also share data on their availability, if this is not an internal organization in the SCT, interfaces should be constructed.
